# 2266. Microbiology and Predictors of Gram-Negative Infections in Persons Who Inject Drugs with Injection-Related Infections Requiring Hospitalization

**DOI:** 10.1093/ofid/ofad500.1888

**Published:** 2023-11-27

**Authors:** Jessica L Mulbah, Kazumi Morita, Laura Mentzer, Sara K Schultz

**Affiliations:** Henry Ford Health, Detroit, Michigan; Temple University Hospital, Philadelphia, Pennsylvania; Temple University Hospital System, Philadelphia, Pennsylvania; Temple University Hospital, Philadelphia, Pennsylvania

## Abstract

**Background:**

Intravenous drug use predisposes users to life-threatening bacterial infections primarily caused by gram-positive organisms. Studies have seen an uptrend in gram-negative injection-related infections in persons who inject drugs (PWID). Therefore, this study aimed to assess the microbiology of injection-related infections in PWID and evaluate risk factors that may predispose these patients to infections caused by gram-negative organisms.

**Methods:**

This retrospective chart review of adult PWID hospitalized with an injection-related infection (skin & soft tissue infection, bacteremia, septic arthritis, endocarditis, epidural abscess, and osteomyelitis) included patients aged >18 years with bacterial growth on specimens collected within 72 hours of admission from September 1, 2021, to March 31, 2022. Data analysis utilized descriptive statistics, chi-square tests, and Mann-Whitney U tests where appropriate.

**Results:**

A total of 259 patients were included in the study. 243 (93.8%) patients grew gram-positive organisms, while only 16 (6.2%) grew gram-negative organisms. The majority of patients were male (60%), the median age was 38 (IQR [33-44]), and 10% had a prior infection with MRSA. The distribution of injection-related infections included SSTIs (79.9%), bacteremia (34.7%), septic arthritis (12%), infective endocarditis (10.4%), osteomyelitis (8.5%), and epidural abscess (3.5%). The most commonly observed organisms were MRSA (36%), *S. pyogenes* (43%), and MSSA (9%). The gram-negative organisms isolated are shown in Figure 1. Approximately 84% of patients received overtreatment with an anti-pseudomonal agent; however, only 2% required its use. SSTIs with lower extremity involvement were found to be associated with gram-negative infections within this cohort, as shown in Table 1.

**Figure 1.**
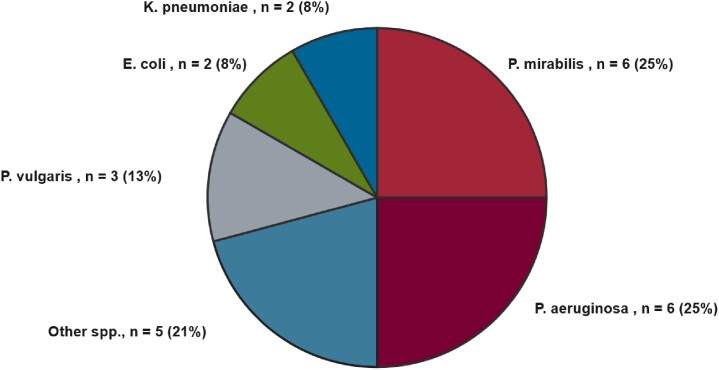
Distribution of gram-negative organisms isolated from cultures collected within 72 hours of admission

**Table 1.**
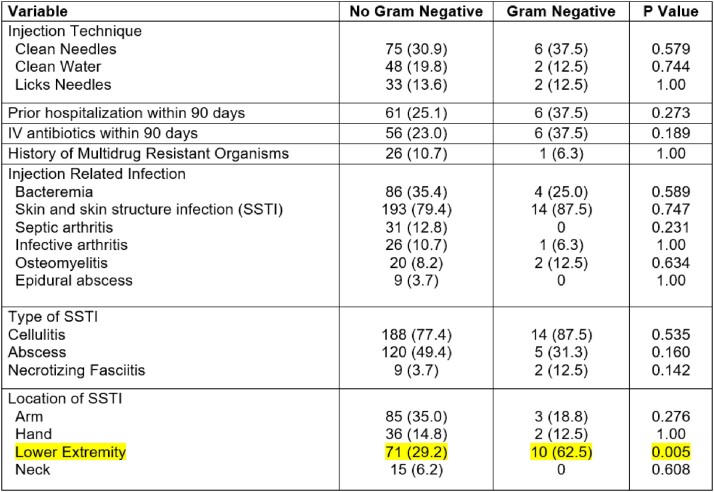
Baseline characteristics and potential risk factors for gram-negative injection-related infections in PWID

**Conclusion:**

In this study, despite less than 10% of patients growing gram-negative organisms on culture, approximately 80% received gram-negative treatment. Knowledge of the microbiology of infections in PWID can aid prescribers in optimizing empiric therapy for injection-related infections and preserving the core principles of antimicrobial stewardship.

**Disclosures:**

**Sara K. Schultz, MD FACP FIDSA**, AbbVie: Advisor/Consultant

